# Strategic Improvements for Gross Anatomy Web-Based Teaching

**DOI:** 10.1155/2012/146262

**Published:** 2011-12-14

**Authors:** David R. Marker, Krishna Juluru, Chris Long, Donna Magid

**Affiliations:** ^1^Department of Radiology, The Johns Hopkins Hospital, 601 North Caroline Street, Baltimore, MD 21287, USA; ^2^Department of Radiology, Weill Cornell Medical College, 525 E. 68th Street, F-056, New York, NY 10065, USA; ^3^The Russell H. Morgan Department of Radiology and Radiological Science, The Johns Hopkins University School of Medicine, 601 N. Caroline Street, JHOC 5165, Baltimore, MD 21287, USA

## Abstract

Current generations of graduate students have been immersed in technology from their early school years and have high expectations regarding digital resources. To better meet the expectations of Gross Anatomy students at our institution, electronic radiology teaching files for first-year coursework were organized into a web site. The web site was custom designed to provide material that directly correlated to the Gross Anatomy dissection and lectures. Quick links provided sets of images grouped by anatomic location. Additionally, Lab and Study Companions provided specific material for the students to review prior to and after lectures and gross dissections. Student opinions of this education resource were compared to student opinions of the prior year's digital teaching files. The new content was ranked as more user friendly (3.1 points versus 2.3 points) and more useful for learning anatomy (3.3 points versus 2.6 points). Many students reported that using the web portal was critical in helping them to better understand relationships of anatomical structures. These findings suggest that a well-organized web portal can provide a user-friendly, valuable educational resource for medical students who are studying Gross Anatomy.

## 1. Introduction

Gross anatomy is a fundamental component of first-year medical school curriculum. During this course, students gain a distinct visual understanding of the organ systems and their relationships to one another. Traditionally this visual understanding has been obtained through a surgical perspective provided by gross dissection or prosection [1]. More recently, with the advent of modern medical imaging, anatomy education has increasingly been supplemented by a radiological perspective [2–4]. Gross Anatomy coursework that provides both perspectives is arguably the ideal training for medical students who will require facility using both views during surgery and radiology rotations or when consulting these services. In support of this educational approach, several recent studies have reported improved clinical training through the use of imaging educational resources [3, 5–7]. In a study that directly compared the approaches, Stanford et al. reported that the combination of gross dissection and computer-based educational tools was a more efficacious teaching approach than either teaching modality alone [8]. 

As imaging has been adopted in the modern medical education, it has benefited from the concurrent development of technologies that have allowed the material to be presented electronically. One of the technologies with the greatest impact has been the Internet [9]. The Internet has increasingly been utilized as an educational tool due to its ability to provide a large volume of educational material in a single, readily-accessible location as well as permitting flexibility in the material format. Images, text, interactive quizzes, and videos can be integrated seamlessly into a comprehensive educational resource. While these capabilities are largely beneficial, they can have a negative effect on the efficacy of the educational resource if the material is poorly organized. For example, if students are confronted with a vast amount of information on the Internet that is presented in a non-user-friendly format, they are likely to either not utilize the resource or not benefit from its use.

One proposed solution for managing Internet material in an organized, user-friendly format is the implementation of a web portal. A web portal is a site that serves as a single point of access to information collected from different sources and presented in multiple formats. Some common features of portals include personalized navigation, for example, “quick links” to frequently accessed information pages, directory-based information structure, community-building tools such as chatrooms, bulletin boards, and emailing lists, user authentication (log in and password), and subject-specific search functionality. Web portals have recently gained in popularity as they have been successfully implemented in a number of clinical settings, such as nursing [10], mental health [11], government disaster preparation [12], patient diabetes information [13], and the MedEdPORTAL implemented by The Association of American Medical Colleges [14]. The general consensus from these studies has been that web portals require initial planning and subsequent maintenance in order to remain relevant and effective but when designed appropriately, this online educational format is robust, easily accessible, and associated with excellent outcomes. While there is limited information regarding the concept of utilizing a portal for medical education purposes, it is reasonable to consider that these benefits recognized in clinically focused web portals would translate effectively for a medical-education-related web portal.

A recent study assessing electronic radiology teaching files for first year Gross Anatomy at our institution suggested the potential need for an improved organization and customization [15]. In its first year, there were 9% of students who reported that they did not find the material user-friendly and over one third of students did not utilize the resource. Based on this assessment as well as a previously defined goal of creating a longitudinal teaching resource for medical student, a decision was made to develop a web portal with several key changes to the existing web content to better organize the educational material. The primary purpose of the present study was to present rational for an approach that was utilized to develop internet-based Gross Anatomy material. Implementation of this teaching resource was assessed by evaluating student opinion regarding its usefulness, determining whether there was any correlation with how the students performed on their anatomy tests and the usage of the web teaching files, measuring student usage patterns of the material, and noting whether there were any design or technical difficulties when developing and managing the web site.

## 2. Materials and Methods

One of the goals of first-year Anatomy curricular redesign efforts at the Johns Hopkins University School of Medicine was to further integrate Radiology into the coursework. The initial efforts for this integration were centered on developing a Medical-Imaging-Resource-Center (MIRC-) based website with relevant teaching files to supplement topics discussed in lecture and corresponding gross dissections [15]. The goal of the present study was to improve on these initial efforts through the creation of a web portal that would organize existing teaching files as well as newly created educational resources and more fully integrate this material into the existing Anatomy coursework.

A survey from the previous year with student feedback from initial efforts in developing Radiology teaching files in a web-based format for Anatomy was reviewed. The team of attending, residents, and medical students working on the second iteration of development used this feedback to identify several possible areas for improvement with the goal for improved user friendliness and increased utilization of the material. Some of the key changes included integration of the material into a web portal, development of Lab and Study Companion components, and a set of organized links to access the material rather than requiring the user to type in search criteria. The list of features that were created and the rational for their implementation are provided in Table 1.

Joomla 1.5 (Open Source Matters, Inc, New York, NY) was utilized as a content management system for creating the web content. Joomla is an open-source application that is freely available on the Internet. It provided the ability to organize and keep track of all content as well as constantly update cases and documents without republishing the web page.

In order to expand the content that would be offered via the web portal, a search for relevant images within the past year was conducted using the Johns Hopkins Hospital Emageon Ultravisual (AMICAS, Inc, Boston, MA) picture archiving and communication system (PACS). There was no copyright protection for these images developed in this iteration of the site development. Although the site was password protected, there was no functionality limiting copying of images from the site by students. This issue will be further addressed in future iterations of the web site development. The effort to obtain several relevant images for each anatomic site was largely in response to student feedback from the prior year that suggested additional imaging teaching files would be beneficial. In order to expand the MIRC content already available, the goal of case acquisition this year was directed towards finding CT and MRI cases where multiple slices could be viewed for each case. Similar to the existing teaching files, the new cases included normal anatomy as well as cases with simple pathology. The cases were initially identified by two of the authors and subsequently reviewed by the senior authors before incorporation into teaching cases. Once selected, the image sets were saved in standard tagged image file format and animated with Macromedia Flash using an open-source student PACS module created by students and residents at the University of Medicine and Dentistry of New Jersey. The new modules allowed users to scroll through images, zoom, and pan while also interacting with labeled structures within the image sets. The decision to utilize Flash was impart based on familiarity of the product by the student developers. In addition, while evaluation of MIRC demonstrated excellent static image teaching file development, it had less robust capabilities compared to the Joomla/Flash combination option for developing more dynamic teaching files with scrolling and highlighting.

The new student PACS modules were combined with last year's MIRC cases to create teaching files that could be accessed during lecture by the instructors and by the anatomy students for review during and following anatomy lectures. MIRC files which were created last year were incorporated into the web site as thumbnails with hyperlinks to the original content, while new thumbnails were created to hyperlink to the students PACS cases. The web portal was designed such that all teaching files were organized by anatomical region. These anatomical regions were defined based on the existing syllabus for the anatomy course. The files were further separated into tutorial files and quiz files. In total, over 100 teaching files were provided (Tables 2 and 3).

**Figure 1:**
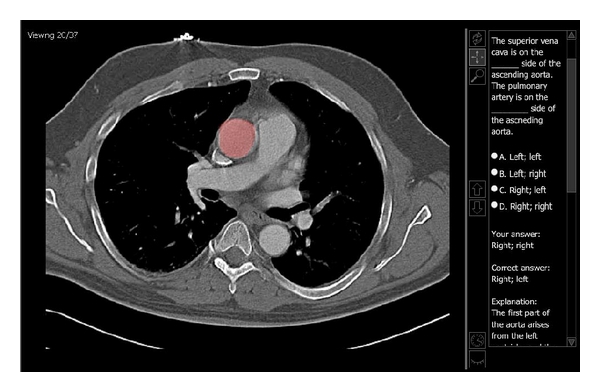
This is a screen-capture of one of the Thorax tutorial files. After clicking on a structure within the image, the structure will highlight and information will be provided about the structure on the right-hand side of the screen. In this example, the arch of the aorta has been selected and information regarding its origin, course, and branches has been provided.

**Figure 2:**
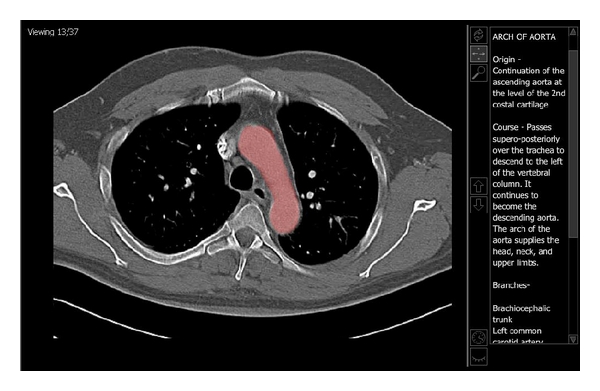
This image illustrates an example of one of the Thorax quiz files. After clicking on a structure within the image, the structure will be highlighted and a question regarding that structure will appear on the right-hand side of the screen. In this example, the ascending aorta has been selected and the correct answer has been chosen displaying an explanation.

In order to create interactive teaching files, relevant anatomy was identified and highlighted on each slice of an image set. A set of questions or relevant teaching points were then linked to each structure so that if the structure was selected on any slice where it could be seen, the questions or teaching points would then appear on the right side of the screen.  Figure 1 displays a sample tutorial case where the arch of the aorta was highlighted and relevant information was displayed for that structure. In Figure 2, a quiz case is displayed where the ascending aorta has been highlighted prompting a multiple choice question related to this structure. For each of the tutorials, there was a page of instructional text, with one associated image set as demonstrated in Figures 1 and 2 and a corresponding set of relevant multiple choice questions.

**Figure 3:**
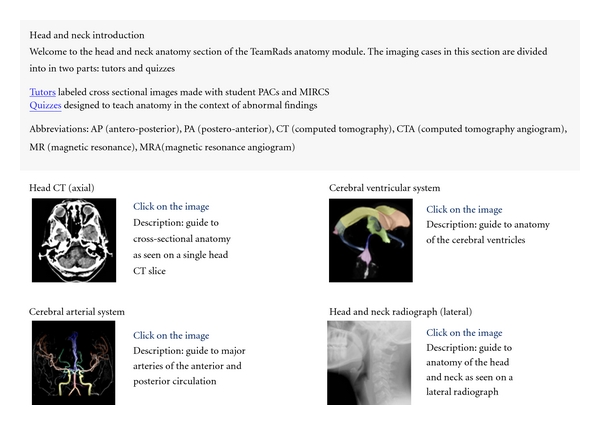
This screen-capture demonstrates the dynamic portion of the web portal home page which provided prelecture material. For this example, the teaching files were provided on the home page a few days prior to the head and neck imaging lecture. The students were expected to review the teaching files prior to lecture.

The web portal was introduced to the medical students on the first day of their anatomy class. The rational for presenting the portal on the first day was to provide students a roadmap to optimize use of Radiology to learn Anatomy and to begin to get them comfortable with the basic layout of the web site. The web address was provided and basic navigation through the site was reviewed. The students were informed that the homepage would be updated a few days prior to each radiology lecture to include pertinent cases (Figure 3). They were required to look over those selected cases prior to lecture and advised to look over the additional cases found in the corresponding anatomical link for further review.

Each radiology lecture was divided into two one-hour segments. During the first hour, the lecturer presented a PowerPoint (Microsoft Corporation, Redmond, WA) lecture on material corresponding to the gross dissection scheduled for that day. The students were then given a five-minute break and asked to close all laptops before the second session began. Next, the lecturer broke the classroom up into small groups based on where the students were sitting and quizzed them on each of the cases found on the homepage of the web portal for that day. This process was repeated for each of the radiology-based lectures so that the students were aware of the expectation that they should be familiar with the corresponding cases found on the web portal homepage.

Following the completion of the Gross Anatomy course (Fall 2010), a fourteen question, web-based survey was distributed to the Anatomy students. The survey was similar to the one distributed to the first-year medical students the previous year (Fall 2009) [15].  Figure 6 provides a complete list of the questions on the survey. In order to evaluate the impact of the web portal compared to the previously available teaching files, the results of the survey from the current year were compared to the results from the previous year.

In order to quantify the utilization of the web site during the anatomy course, a StatistX module was installed into the Joomla content manager. This module allowed a hit counter to be incorporated into the web site which would keep track of all web traffic received by the site. The module provided daily activity, hourly reports, top ten pages hit, and the last twenty pages visited. This module provided information regarding when the students were utilizing the website the most and what content they were looking at. IP addresses were also provided in the statistics indicating whether the students preferred to access the material on or off campus. As previously noted, the teaching files were integrated to a greater extent into the coursework by including the material in discussion groups rather than the lecture-only format utilized during the previous year. The server data from the current year was compared to the server data from the previous year to identify any changes in the student utilization patterns after these changes in the structure of the coursework.

## 3. Statistical Analysis

After collecting the responses to the web-based survey, the data was exported from the survey site (http://surveymonkey.com) to spreadsheet format for aggregating and graphing the data. The spreadsheet data was imported into SPSS version 13.0 software (SPSS, Chicago, Illinois) for additional data analysis and statistical calculations. Comparisons of proportions for student responses for the current year to the prior year were made utilizing a chi-square analysis with a Yate's correction. A Mann-Whitney rank sum test was conducted to compare the mean values of the student responses. *P* values less than 0.05 were considered statistically significant.

## 4. Results

The demographic profile of the students who responded to the web-based survey this year was similar to the prior year. There were 87 of 120 medical students (73%) who completed the survey this year compared to 71% who completed the survey the previous year (*P* = 0.886). In both cohorts, the majority of the students was between 22 and 25 years old and had an undergraduate degree in biology. The only difference in demographics noted between the two groups was that the more recent cohort of students had a smaller percentage of Fine Arts majors (5% compared to 15%, *P* = 0.036).

Compared to the previous electronic teaching files, the new content received better evaluations for user friendliness and usefulness (Table 4). The mean score for usefulness of the web portal content was 3.3 points (helpful to very helpful) compared to 1.5 points (not helpful to somewhat helpful) for the previous material (*P* < 0.001). When excluding the 0 point scores of the students who did not use the online teaching files last year, the mean score improved from 1.5 to 2.6 points (somewhat helpful to helpful). However, the 2.6 points remained statistically less than the 3.3 point rating for the web portal (*P* < 0.001). Similarly, the new web material was rated as being more user friendly 3.1 points (good to excellent) versus 2.3 points (satisfactory to good) even after correcting again for the students who did not use the online content (*P* < 0.001).

**Figure 4:**
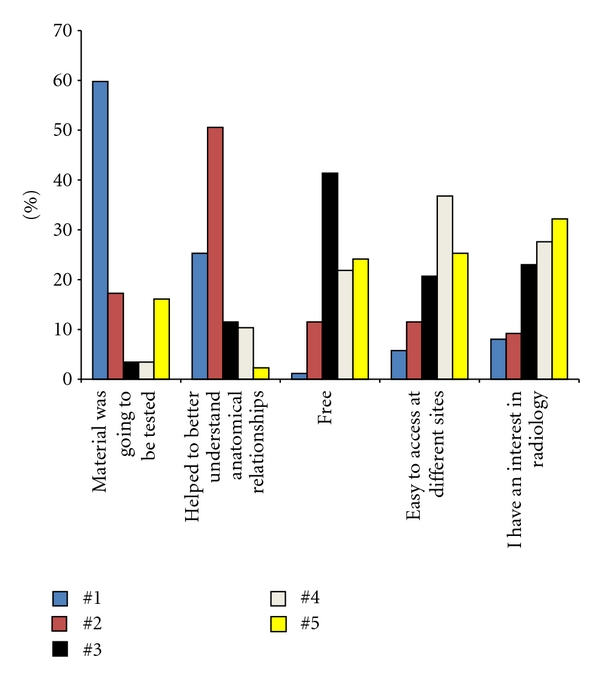
This bar graph illustrates the responses of the students regarding their ranking of why they utilized the Radiology teaching files.

The primary reason students utilized the resources was to review material that was going to be tested (Figure 4). There were 52 (60%) students who ranked this as the number one reason for accessing the teaching files, and 67 (77%) students ranked it as either the no. 1 or no. 2 reason they used the web material. The number of students who indicated that the teaching files were used because they helped to better understand anatomical relationships was only slightly less with 66 (76%) students ranking this option as either their no.1 or no. 2 choice. There were few students who indicated that their primary reason for using the teaching files was because they were free. However, many students ranked this as their second or third reason for utilizing the site, and this was the third highest ranked choice overall.

**Figure 5:**
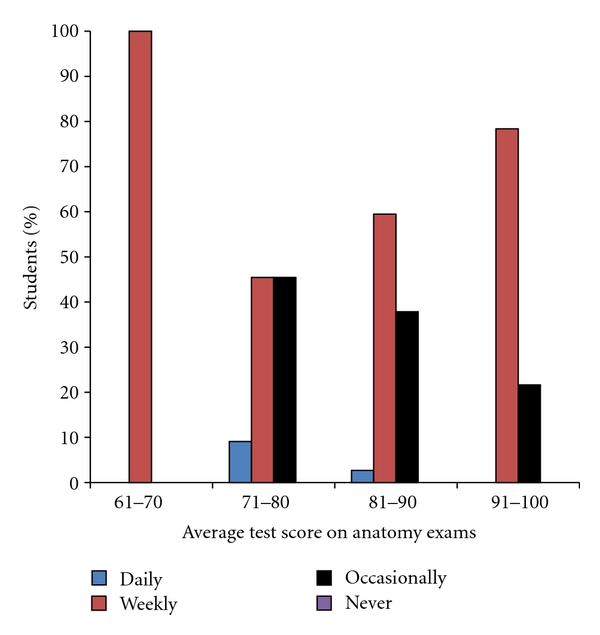
This bar graph groups the students by the performance on the Gross Anatomy exams and depicts how frequently they utilized the Radiology teaching files.

**Figure 6:**
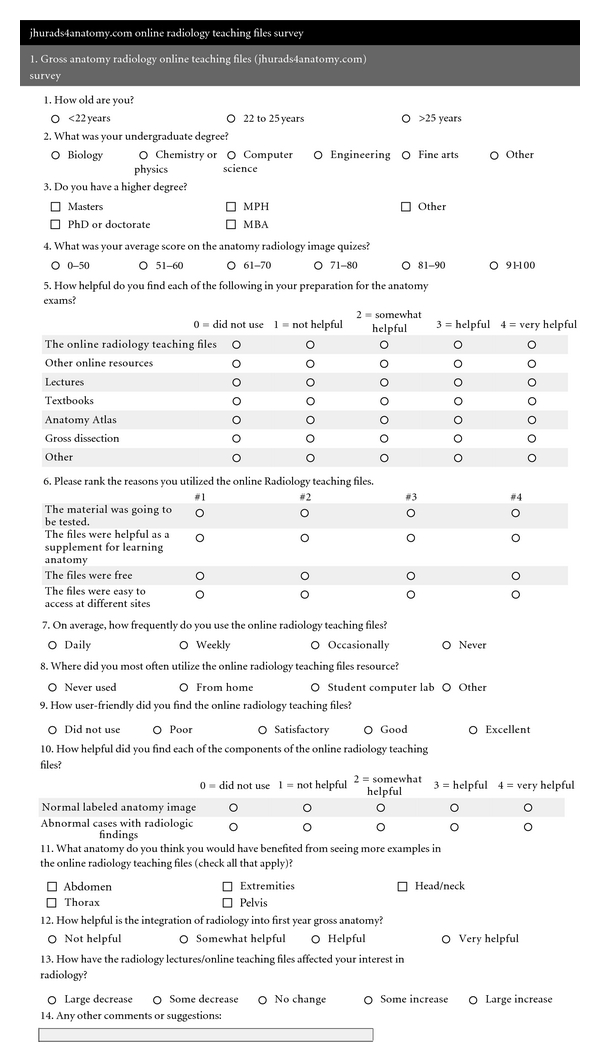


The numbers of students in each of the test score categories were 2 students with average scores 61 to 70, 11 students with scores 71 to 80, 37 students with scores 81 to 90, and 37 students with scores 91 to 100. There appeared to be a trend for the highest scoring students (91 to 100 averages) to utilize the web portal more frequently than the 71-to-80 and 81-to-90 groups (Figure 5). However, there were insufficient numbers of students in the various test score groups to perform a chi-square analysis with any sufficient degree of certainty.

There were no students who completed the survey that did not use the radiology-based teaching materials available on the web site. This finding was consistent with required prelecture study files and subsequent review files that were provided as study aids for similar material that would be tested. The web server traffic pattern demonstrated a baseline of 30 to 40 hits per day. The number of hits increased to over 100 during the two to three days prior to an exam.

There were minimal technical or design difficulties in the creation and maintenance of the web portal. After being introduced to the web portal on the first lecture, no students reported any difficulty accessing the Radiology teaching files from home or at the school computer labs. Trials of accessing the site with various web browsers, including Internet Explorer 8, Safari 4, Flock 2, Chrome 3, Opera 10, Netscape Navigator 9, and Firefox 3.5, demonstrated no loss of functionality. There were no instances where the server went down throughout the course. At the end of the course, there were multiple students who suggested that the functionality of the online PACS modules needed to be improved. Students expressed frustration with the fact that the modules required the student to identify a structure correctly before moving to the next question. For example, one student noted that “It would be really helpful if the online modules allowed you to get a hint if you cannot find a structure. The way the quizzes are set up currently, if you cannot find something, you just have to skip it and move onto something else. The CTs would be much more helpful if it were possible to view an index of all of the labeled items.” Similarly, another student is quoted as saying, “The website is a good start. I would want a system where the different parts of the radiographs are already labeled so that I do not have to randomly click around the image to find one tiny structure.” Another area the students suggested for improvement was to add even more teaching files. 74 (85%) of the students indicated that they would benefit from more cases from one or more anatomic site. The most common sites indicated were head and neck (*n* = 65, 88%) and extremities (*n* = 38, 51%).

## 5. Conclusions

Recent student feedback at our institution suggested the need for a more organized approach for presenting the Radiology component of the first year medical school Gross Anatomy course. Based on feedback from earlier efforts and assessment of studies that have shown successful approaches for implementing web portals for clinical practice, efforts were made to better organize Radiology teaching files prepared for Gross Anatomy. In general, the results of the present study suggest that organizing web material to better correspond to Anatomy course work and providing it in a structured set of links in a web portal was associated with increased student satisfaction and utilization of the resource.

One of the factors that may have facilitated the development of a useful tool for Gross Anatomy students is that some of the authors are medical students or are only a few years removed from taking the course themselves and can relate to what material may be useful for Gross Anatomy study. This hypothesis is supported by a recent study by Rosenbaum et al. [16]. They evaluated medical student involvement in the development of a website to act as each individual medical student's homepage. They created a web portal that provided students with access to course material, evaluations, academic information, and community assets. Based on greater than 80% positive feedback regarding the web portal, they recommended that other medical schools that are creating and expanding digital resources should solicit the valuable input and perspective of medical students.

It was and will be difficult to analyze the effect of the web portal on student performance since it was felt to be inappropriate to withhold access from half the class for a controlled comparison. Our students are highly motivated and sophisticated learners, as well as “digital natives” accustomed to using web resources; it was felt their comments and satisfaction scores were legitimate metrics of utility or the resource. There also continue to be changes in the overall Gross Anatomy course structure and student testing reflecting as our medical school's new curriculum moves through its second year, further complicating comparison of test scores or grades from the initial year of web site usage to the second year with the improved/modified web material.

Although the primary objective for the present study was to create user-friendly teaching files for anatomy students, the ultimate goal of the Radiology research team working on this project was to create the foundation for a medical student and resident Radiology education web portal. The concept for the web portal was derived from previous studies at our institution that have highlighted the benefit of longitudinal directed teaching [7, 17]. For example, Feigin et al. reported that recall and retention toward the end of medical school was facilitated by providing preclinical instruction followed by review of the material later in school [7]. They reported that senior students benefited from previously received preclinical radiology training as they were able to improve their score on an anatomy quiz from an average of 4.42 (standard deviation 1.34) to a score of 8.65 (standard deviation 1.24) only three weeks later. The goal for the web portal in the present study is to provide a similar benefit but in an electronic format. The web portal design updates that have now been included in an effort to meet this goal are components for (1) imaging modules to be integrated into the basic clerkships (PRECEDE Modules for third year medical students), (2) lectures from The Johns Hopkins Basic Radiology Elective (for third- and fourth- year medical students), and (3) Johns Hopkins Radiology Resident Joint Procedure notes (for residents). For those students interested in pursuing a career in radiology or just looking for residency match advice, a residency application guide (*APPS of STEEL)* created by the senior author was also included. Finally, links to other recommended anatomy and radiology resources were incorporated into the site to allow students and residents to further their interest in the field.

While all of this functionality of the portal was customized for a single institution, the authors are assessing possible collaborations with other institutions. There is a potential to provide a more universal product. This product could potentially be provided to international colleagues who may not have the funds to develop a similar resource. To provide this outreach, it is conceivable that such a product could linked to be included under the umbrella of a larger entity with greater outreach such as the MedEdPORTAL.

The students indicated that the number one reason for accessing the files was to prepare for the material possibly being on the test. Although this may have been the primary incentive, it is important to note that regardless of the reason the students accessed the material initially, there were a large number of students who subsequently found the material to be user friendly and helpful in learning to identify anatomic structures. Furthermore, it is reasonable to expect students who are required to utilized material that will be tested to be more hypercritical of the material. As such, the improvements in reported user-friendliness of the electronic material are arguably more substantial. Of interest, students constantly came forward or emailed us during the semester to make constructive suggestions or to inquire about joining the project, also implying engagement. These findings suggest that Educators should recognize that by informing students that material will be on their tests, a majority if not all of the student body will review the resource. The goal of the Educator, however, should not only be to have students utilize the resource but to also provide material that is user friendly and provides optimal educational value. If Educators are considering a digital format for distributing material, a structured web site can be a tool to provide easy access, in a user friendly format.

Similar to other questionnaire-based studies, one of the limitations of the present study is the number of students who do not participate. As previously noted, the response rate of 73% for the present study was similar to the rate for the survey from the previous year. This response rate is approximately one standard deviation above the normal response rates reported in the literature [18]. While it is uncertain how the remaining 27% of students would have answered the survey questions, we believe that based on the reported demographics and test scores that the study population was a fair representation of the class on a whole. Another limitation is that the survey was not formally validated prior to online implementation. However, students did not report problems when completing the survey, and standard survey functionality provided by the vendor was utilized to ensure students completed all required questions. One other limitation was the accuracy of the self-reported test scores. However, the range of scores reported appeared to reflect the class actual performance and with no identifiers for the responses the students had no incentive to not report their true performance.

In summary, this study suggests that students have a favorable impression of having a web site with organized Radiology resources for first-year Gross Anatomy. We are encouraged by the increased utilization of this resource compared to the resources provided last year. There remains room for improvement with students suggesting that they would benefit from more teaching files and from modules that allow for a summary view rather than requiring point by point walk through of the material. Based on the first two years' experience and survey feedbacks, and our observations and increasing experience, we will further expand and refine this teaching resource before the 2011 Gross Anatomy course and hope to be able to further explore and assess the initial trends and findings discussed here. Additional studies are being planned that will further assess other components of the web portal and the efficacy of the site as a longitudinal educational resource for students throughout medical school.

## Figures and Tables

**Table 1:** Rational and features of newly created Radiology web portal for Gross Anatomy.

Goal/Rational	Feature	Description
Provide multiple formats for viewing and learning Radiology.	Utilized MIRC, Flash, and Joomla for Creating Teaching Files	MIRC provides excellent functionality for developing static single image teaching files. Macromedia Flash using an open-source student PACS module and Joomla content management system readily allowed for creation of teaching files with multiple-level cross-sections, and animations.
Provide single site for Radiology teaching material that students will utilize starting day one Gross Anatomy and then throughout medical school.	Home Page	Structured Web Portal with an integrated anatomy component.
Begin to expose students to clinical application of anatomy.	Cases of the Week	Clinical cases with relevant findings on imaging updated on homepage to correspond to lectures.
Provide students with direct correlations between cadaver anatomy and imaging.	Laboratory Companion	Feature of Web Portal providing imaging relevant to the day's dissection.
Provide content that allows students to identify labeled anatomy on imaging.	Study Companion	Feature of Web Portal providing labeled images and cross sectional modules.
Ensure students recognize reasoning for integrating Radiology and Anatomy.	Module Goals	Clearly stated goals provided on home page.
Provide students with a roadmap to optimize use of Radiology to learn Anatomy.	Methods for Achieving Goals	Discussed in lecture on first day of class and clearly stated on home page.
Allow Radiology files to be reviewed by anatomic site.	Organization of Files	Students click links that instantly bring up files organized by anatomic site.
Facilitate web site navigation by giving quick views of available material.	Preview of Teaching Files	Students click thumb nail with an image preview as well as text description.
Develop site that will serve as a platform for Radiology learning throughout medical school.	Longitudinal Educational Components	Web Portal provides additional components for basic Medicine and Surgery clinical rotations.
Reflect importance of cross-sectional imaging in clinical medicine.	Cross Section Teaching Files	Static images plus new cross section files that allow scroll, pan, and zoom.
Provide lecture presentations for preview and review of material.	Lecture material	Lecture PowerPoints made available via link on the Web Portal.
Introduce students to other existing resources for learning Radiology and Gross Anatomy.	Additional Learning Tools	Information provided in syllabus and links to resources provided on the web portal.
Introduce students to advancements being made in Radiology which may have future clinical implementation.	Anatomy TV	3D video anatomy teaching files available

**Table 2:** Characterization of teaching files by question type.

Anatomical region	Tutorials	Quizzes	Normals	Abnormals
Thorax	11	18	15	14
Abdomen	7	22	11	18
Pelvis	3	7	7	3
Limbs	9	18	10	17
Spine	1	2	1	2
Head and neck	2	4	5	1

Total	33	71	49	55

**Table 3:** Characterization of teaching files by imaging modality.

Anatomical region	CT	MRI/MRA	US	Plain film	Angiography (Fluoroscopy)
Thorax	9	3	0	15	2
Abdomen	14	0	1	11	3
Pelvis	6	0	0	4	0
Limbs	1	0	0	25	1
Spine	0	0	0	3	0
Head and neck	1	4	0	1	0

Total	31	7	1	59	6

**Table 4:** Comparison of student opinion of Radiology Teaching Files from Fall 2009 versus Fall 2010.

Data	Searchable Database (2009)	Web Portal (2010)	*P* value
*Helpful in exam preparation?*			<0.001*
Very helpful	8 (9%)	40 (46%)	
Helpful	16 (19%)	36 (41%)	
Somewhat helpful	22 (26%)	9 (10%)	
Not helpful	4 (5%)	2 (2%)	
Did not use	35 (41%)	0 (0%)	

*user-friendly?*			<0.001*
Excellent	3 (4%)	28 (32%)	
Good	16 (19%)	41 (47%)	
Satisfactory	23 (27%)	16 (18%)	
Poor	8 (9%)	2 (2%)	
Did not use	35 (41%)	0 (0%)	

*Statistically significant.

## References

[1] Winkelmann A (2007). Anatomical dissection as a teaching method in medical school: a review of the evidence. *Medical Education*.

[2] Choi ARA, Tamblyn R, Stringer MD (2008). Electronic resources for surgical anatomy. *ANZ Journal of Surgery*.

[3] Erkonen WE, Albanese MA, Smith WL, Pantazis NJ (1992). Effectiveness of teaching radiologic image interpretation in gross anatomy: a long-term follow-up. *Investigative Radiology*.

[4] McNiesh LM, Madewell JE, Allman RM (1983). Cadaver radiography in the teaching of gross anatomy. *Radiology*.

[5] Jafri NF, Wu P, Stanfield L, Slanetz PJ (2008). Use of radiologic imaging to enhance physical diagnosis instruction in the preclinical curriculum. *Academic Radiology*.

[6] Petersson H, Sinkvist D, Wang C, Smedby O (2009). Web-based interactive 3D visualization as a tool for improved anatomy learning. *Anatomical Sciences Education*.

[7] Feigin DS, Magid D, Smirniotopoulos JG, Carbognin SJ (2007). Learning and retaining normal radiographic chest anatomy. Does preclinical exposure improve student performance?. *Academic Radiology*.

[8] Stanford W, Erkonen WE, Cassell MD (1994). Evaluation of a computer-based program for teaching cardiac anatomy. *Investigative Radiology*.

[9] Richardson ML (1995). A World-Wide Web radiology teaching file server on the Internet. *American Journal of Roentgenology*.

[10] Campbell TB (2009). Role socialization: designing a web-based program to orient new school nurses. *Journal of School Nursing*.

[11] Christensen H, Murray K, Calear AL, Bennett K, Bennett A, Griffiths KM (2010). Beacon: a web portal to high-quality mental health websites for use by health professionals and the public. *The Medical Journal of Australia*.

[12] Bader JL, Nemhauser J, Chang F (2008). Radiation event medical management (REMM): website guidance for health care providers. *Prehospital Emergency Care*.

[13] Nordqvist C, Hanberger L, Timpka T, Nordfeldt S (2009). Health professionals’ attitudes towards using a web 2.0 portal for child and adolescent diabetes care: qualitative study. *Journal of Medical Internet Research*.

[14] Reynolds RJ (2008). MedEdPORTAL: educational scholarship for teaching. *Journal of Continuing Education in the Health Professions*.

[15] Marker DR, Bansal AK, Juluru K, Magid D (2010). Developing a radiology-based teaching approach for gross anatomy in the digital era. *Academic Radiology*.

[16] Rosenbaum BP, Gorrindo TL, Patel SG, McTigue MP, Rodgers SM, Miller BM (2009). Medical student involvement in website development. *Medical Teacher*.

[17] Magid D, Hudson DW, Feigin DS (2009). Chest radiographic anatomy retention. The impact of preclinical groundwork on clinical recall in two schools. *Academic Radiology*.

[18] Baruch Y, Holtom BC (2008). Survey response rate levels and trends in organizational research. *Human Relations*.

